# Spatiotemporal characteristics of gaze of children with autism spectrum disorders while looking at classroom scenes

**DOI:** 10.1371/journal.pone.0175912

**Published:** 2017-05-04

**Authors:** Takahiro Higuchi, Yuko Ishizaki, Atsushi Noritake, Yoshitoki Yanagimoto, Hodaka Kobayashi, Kae Nakamura, Kazunari Kaneko

**Affiliations:** 1 Department of Pediatrics, Kansai Medical University, Hirakata, Osaka, Japan; 2 Department of Physiology, Kansai Medical University, Hirakata, Osaka, Japan; Medical Photonics Research Center, Hamamatsu University School of Medicine, JAPAN

## Abstract

Children with autism spectrum disorders (ASD) who have neurodevelopmental impairments in social communication often refuse to go to school because of difficulties in learning in class. The exact cause of maladaptation to school in such children is unknown. We hypothesized that these children have difficulty in paying attention to objects at which teachers are pointing. We performed gaze behavior analysis of children with ASD to understand their difficulties in the classroom. The subjects were 26 children with ASD (19 boys and 7 girls; mean age, 8.6 years) and 27 age-matched children with typical development (TD) (14 boys and 13 girls; mean age, 8.2 years). We measured eye movements of the children while they performed free viewing of two movies depicting actual classes: a Japanese class in which a teacher pointed at cartoon characters and an arithmetic class in which the teacher pointed at geometric figures. In the analysis, we defined the regions of interest (ROIs) as the teacher’s face and finger, the cartoon characters and geometric figures at which the teacher pointed, and the classroom wall that contained no objects. We then compared total gaze time for each ROI between the children with ASD and TD by two-way ANOVA. Children with ASD spent less gaze time on the cartoon characters pointed at by the teacher; they spent more gaze time on the wall in both classroom scenes. We could differentiate children with ASD from those with TD almost perfectly by the proportion of total gaze time that children with ASD spent looking at the wall. These results suggest that children with ASD do not follow the teacher’s instructions in class and persist in gazing at inappropriate visual areas such as walls. Thus, they may have difficulties in understanding content in class, leading to maladaptation to school.

## Introduction

Autism spectrum disorders (ASD) is a neurodevelopmental disorder marked by impairments in social interaction and communication, as well as repetitive and restricted behaviors [1]. Children with ASD, even those with average or high intelligence, often refuse to go to school because of emotional distress caused by their increased sensitivity to stimuli, delayed learning, and lack of understanding of their characteristics by others [2–4]. However, because children with ASD who have average or high intelligence may seem to have normal verbal communication, the diagnosis of ASD is often delayed. Thus, early diagnosis and intervention in such children is critical to improve their quality of life.

Measurement of eye movements is a promising tool for diagnosing and assessing children with ASD. The noninvasive nature of this method is particularly beneficial for children with ASD, because most of these children have increased sensitivity to noise (hyperacusis) or touch (tactile hyperesthesia). This is particularly true for young children and infants with ASD, who have low verbal ability. Previous studies of gaze behavior of children with ASD [5, 6] using visual stimuli such as social scenes from a film [7], human faces [8], and geometric figures in static and dynamic images [9, 10] showed that the children spent more time looking at whole-image and nonsocial stimuli than did children with typical development (TD). Children with ASD also tend to look at irrelevant targets [11], even when they are instructed to detect and name targets that others are looking at [12]. These results suggest that children with ASD have difficulty in sharing focus on an object with other individuals, which is known as joint attention, because they are poor at maintaining eye contact [13, 14]. The greater dispersion of gaze points in children with ASD might be jumped from social cues [15], which makes it difficult for the children to obtain information from others in daily life.

Parsons reported that school classes and relationships with class members are similar to a social system [16]. The skills required to understand a teacher’s instructions include joint attention and communicating with the teacher, and students need to fit into the society to function well in school. Even though children spend much time at school, the gaze behavior of children with ASD in actual classroom scenes remains poorly explored. In this study, we investigate gaze characteristics of children with ASD by using actual classroom scenes.

We predict that children with ASD spend less gaze time looking at a teacher’s face and finger, whereas they spend more time looking at geometric figures drawn on a blackboard in the classroom scenes. They also spend less time looking at objects pointed at by a teacher because of less joint attention. These behavioral characteristics in the classroom persist even in the higher grades, and therefore, children with ASD have difficulty in understanding the contents of lessons in the classroom.

## Materials and methods

### Participants

Fifty-three children participated in this study: 26 children with ASD (19 boys and 7 girls; mean age, 8.6 ± 3.3 years; range, 3 to 14 years) and 27 age-matched children with TD (14 boys and 13 girls; mean age, 8.2 ± 3.9 years; range, 2 to 15 years). The participants were recruited from the Kansai Medical University Medical Center. Characteristics of the participants are given in Table 1.

**Table 1:** Characteristics of the participants.

Mean (SD) [Range] *P* value			
	ASD	TD	ASD vs TD
Characteristics	(n = 26)	(n = 27)	
Sex, M/F	19/7	14/13	0.16
Age	8.6 (3.3) [3–14]	8.2 (3.9) [2–15]	0.40
IQ or DQ	92 (13.7)		
CARS score	27.9 (3.8)		
PARS-TR peak score	20.5 (4.4)		
PARS-TR current score	21.3 (8.1)		

Abbreviations: ASD, autism spectrum disorder; TD, typical development

IQ, intelligent quotient; DQ, developmental quotient

CARS, Childhood Autism Rating Scale; PARS-TR, Parent-interview ASD Rating Scale—Text Revision.

The children were regarded as those with TD by specialists in developmental pediatrics when they met the following inclusion criteria: i) absence of past and present histories of chronic illness; ii) absence of developmental and/or psychiatric problems indicated at the health and developmental check-up when they were 3 years old; and iii) absence of special educational needs.

The inclusion criteria for children with ASD were as follows: i) the children met the criteria for autistic disorder or pervasive developmental disorder according to the *Diagnostic and Statistical Manual of Mental Disorders*, fourth edition, text revision (DSM-IV-TR)[1]; ii) the intelligence quotient (IQ) of children aged >5 years based on the Wechsler Intelligence Scale for Children, fourth edition (WISC-IV) [17], or the developmental quotient (DQ) of children aged ≤5 years based on the Kyoto Scale of Psychological Development, whose DQ is strongly correlated with IQ [18], were more than 70; iii) the scores of Childhood Autism Rating Scale (CARS) [19] and/or the Parent-Interview ASD Rating Scale, text revision (PARS-TR) [20], were above their cut-off value.

The exclusion criteria for children with TD and those with ASD were as follows: i) presence of any psychiatric illness in the past or present histories; ii) presence of problems in eyeball movement or visual function; iii) inability to accomplish a 10-min experiment.

#### Developmental assessment

We also assessed the children with ASD using the Childhood Autism Rating Scale (CARS) [19] and/or the Parent-Interview ASD Rating Scale, text revision (PARS-TR) [20]. The CARS score, which ranges from 15 to 60, indicates the degree of autism of a child: less than 30 indicates no autism, 30 to 37 indicates mild to moderate autism, and over 37 indicates severe autism. Tachimori et al. [21] recently reported that children diagnosed with Asperger’s syndrome who had IQs in the average or high average range in addition to ASD characteristics were differentiated from those without Asperger’s syndrome by cutoff values of 25.5/26.0. Applying this criterion, we also used the cutoff values of 25.5/26.0 to differentiate children with ASD from those with TD, taking into account the DQ or IQ of our children with ASD. The mean CARS score of our participants who were diagnosed as ASD was 27.9 ± 3.8 (standard deviation), which met Tachimori’s criterion of Asperger’s syndrome. The PARS-TR in Japan (PARS Committee, 2013) involves evaluation of ASD symptoms by a semi-structured interview with a parent or family member of a subject. It comprises both an evaluation of current symptoms (the current symptoms scale) and that of the most pronounced symptoms during infancy (the peak symptoms scale). There was a significant correlation between the PARS-TR scores and the Autism Diagnostic Interview-Revised (ADI-R) scores, particularly between the Qualitative Abnormalities in Reciprocal Social Interaction in the ADI-R score and the Social Communication in the PARS-TR score [22]. In this study, the mean PARS-TR peak symptoms score was 20.5 ± 4.4 and the mean current symptoms score was 21.3 ± 8.1. These scores are much higher than the cutoff values for PARS-TR (9/10). Taken together, these scores confirmed the ASD diagnosis of the participants in our study.

The experiment was conducted in a quiet, well-lighted room in Kansai Medical University Medical Center. A partition separated the participants from our operators and the experimental apparatus. The participants were instructed to freely watch silent movies of actual classroom scenes on a 48 × 30 cm^2^ monitor. The monitor was placed approximately 60 cm away from the participants’ eyes, with its center positioned at eye level. While watching the movies, the children sat on their parent’s lap or on a chair with their chin on a chin rest to stabilize the position of their head. The total time of the experiment was less than 10 min.

Gaze positions were measured at 250 Hz with a remote eye tracker (iView X RED eye tracking system, SensoMotoric Instruments, Teltow, Germany). An infrared source and a camera were set below the monitor.

### Experimental stimuli

**Fig 1:**
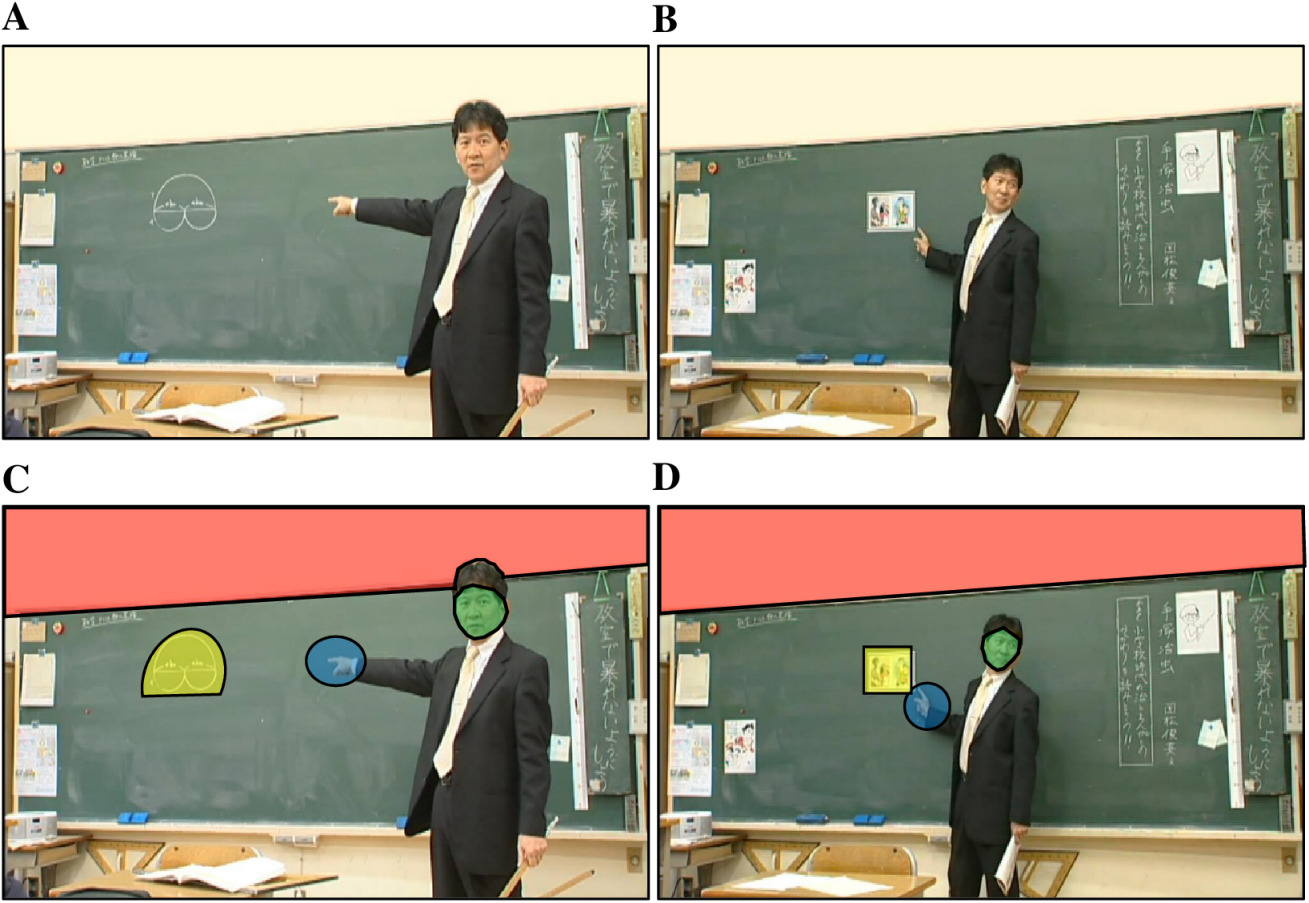
Representative stimuli in the arithmetic and Japanese classroom scenes. (A) The teacher is pointing to the geometric figure in the arithmetic class. He is standing far from the figure, and his eyes are directed to a participant. (B) The teacher is pointing to the cartoon characters in the Japanese class. He is standing near the characters, and the direction of his eyes is incongruent with that of his finger. (C, D) The green ROIs represent the teacher’s face, the blue ROIs represent the teacher’s fingers, the yellow ROIs represent the figures at which the teacher is pointing, and the red ROIs represent the wall with no objects.

The stimuli used in this experiment were short movies of actual classroom scenes at an elementary school, which were recorded with the permission of the students, their parents, the teacher, and the schoolmaster and approved by the Katsuragi Municipal Board of Education and Taima Elementary School in Nara Prefecture, Japan, for research use only. The movies showed a class in Japanese and a class in arithmetic. In the Japanese classroom scenes, a teacher pointed at cartoon figures of a person’s face and body drawn on the blackboard. In the arithmetic classroom scenes, he pointed at geometric figures (Fig 1A and 1B). The distance from the teacher’s finger to the objects at which he was pointing was categorized as “near” or “far,” and the direction of the teacher’s gaze was categorized as toward the participants (“direct”) or toward (“congruent”) or away from (“incongruent”) the objects at which he was pointing. Thus, the combination of two distances from the objects and three directions of the teacher’s gaze gave a total of six situations for the classroom scene. The classroom scenes of different subjects (Japanese and arithmetic) were presented separately in different blocks. The order of the movies in a given block was counterbalanced across the participants.

### Measurements of gaze pattern

The experiment began with an eye-positioning calibration procedure in which the participants were required to fixate on a shrinking red circle that was presented sequentially at different positions on the monitor. Subsequently, a set of six short movies, each lasting for 5 s and separated by a 2-s gap, was shown sequentially at the center of the monitor. The length of each movie was determined so that the participants could attend long enough to furnish sufficient data for the analyses. Between the short movie presentations, correction of eye position was performed by requiring the subjects to fixate on a blinking white circle. This procedure was crucial since the head positions of both the ASD and TD subjects could be unstable during the experiments. There was a 30-s interval between the Japanese and arithmetic classroom scenes. The eye-tracking data were processed with custom software written in MATLAB (Mathworks, Natick, MA, USA).

### Ethical approval

After the experimental procedures had been explained to the participants and their parents, we confirmed their willingness to participate in the study and obtained written informed consent from the participants or their parents. The research was approved by the Ethics Committee of Kansai Medical University (No. 1100). The experiment was conducted from July 2014 to September 2016.

## Results

### General gaze tendency and setting of the regions of interest

**Fig 2:**
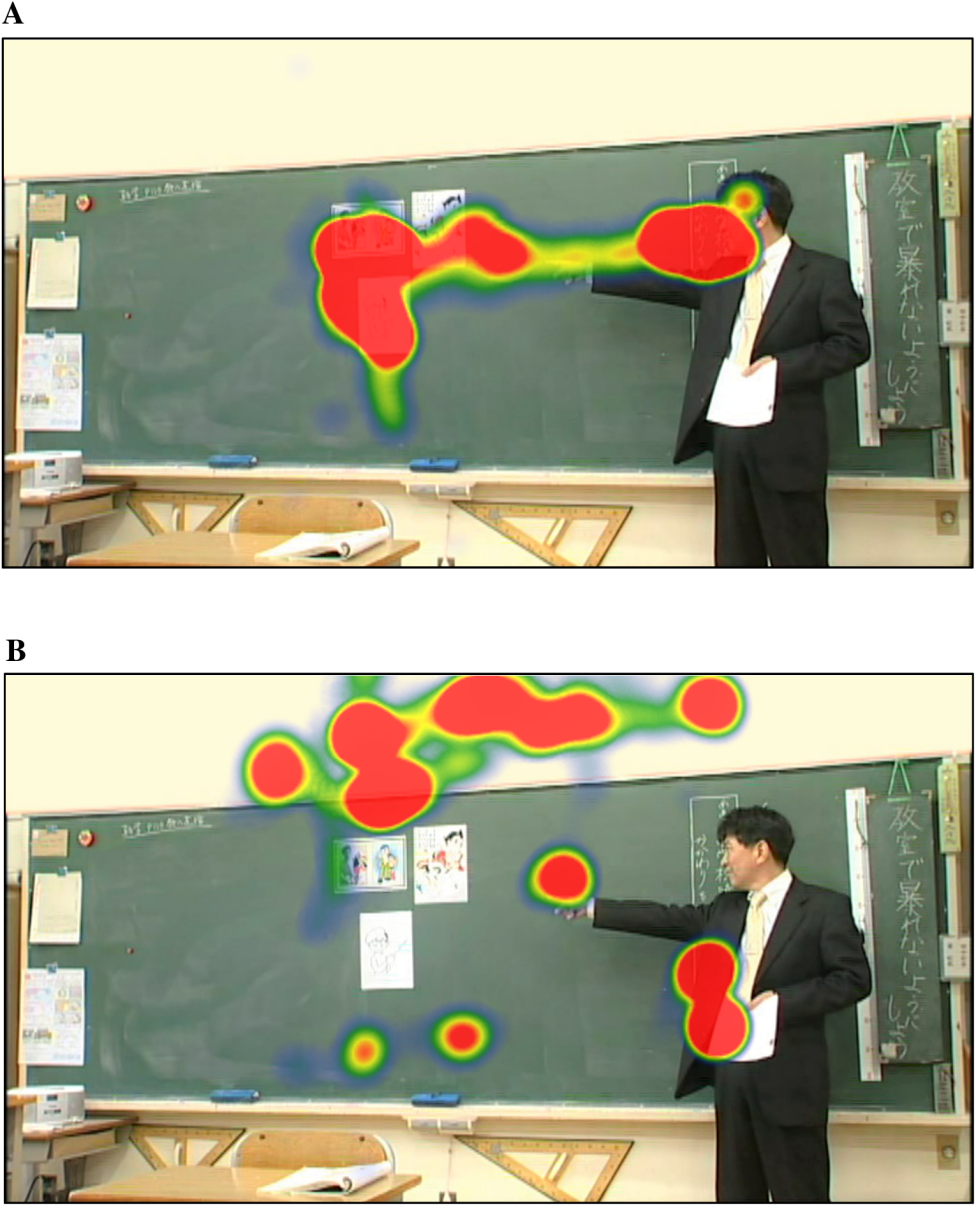
Representative heat-map figures. Representative still frame used to show the heat maps of a child with TD and another with ASD. Red areas are gazed at by children for longer periods. (A) Heat map of a child with TD. (B) Heat map of a child with ASD.

Fig 2 shows the example of gaze pattern of a child with TD (Fig 2A) and another with ASD (Fig 2B) represented by heat-map plots. Both children gazed at the teacher’s fingers and face, the figures on the blackboard pointed at and/or looked at by the teacher, and a wall surrounding the blackboard. To quantify the spatiotemporal pattern of gaze behavior in children with ASD and those with TD, we first identified these visual areas as regions of interest (ROIs) (Fig 1C and 1D). The blue area represents the teacher’s finger, the green area represents the teacher’s face, and the yellow area indicates the object at which he is pointing on the blackboard, all of which are important for understanding the classroom lesson. The red area, which was the wall with no objects, was irrelevant to the lesson.

### Proportions of gaze time on each ROI

**Fig 3:**
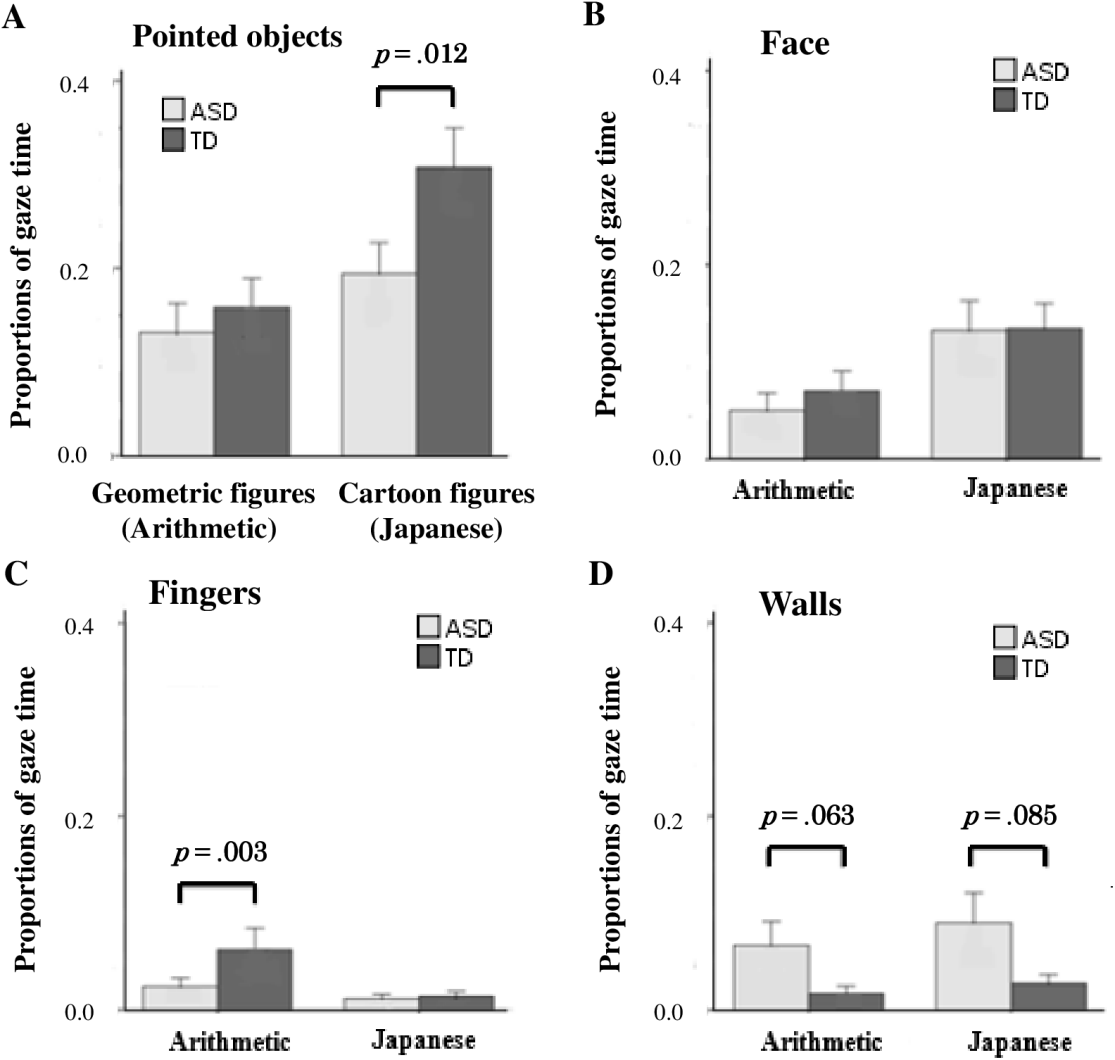
Proportions of gaze time for the two groups on each ROI in each classroom scene. The proportions of gaze time for the two groups (mean ± standard error of mean) are shown. The geometric and cartoon figures pointed at by the teacher (A), the teacher’s finger (B), the teacher’s face (C), and the walls, not objects, pointed at by the teacher (D) in the arithmetic and Japanese classroom scenes. Gray bars indicate children with ASD, and dark gray bars indicate children with TD. *P* values were determined by Welch’s *t*-test.

The whole series of movies lasted for 30 s (5 s for each of the six movies). During this 30-s period, we first measured the total viewing time, defined as the time when the position of the eyes was within the 48 × 30 cm^2^ screen. The total time of watching class scene movies did not significantly differ between the children with ASD and those with TD (arithmetic class: mean ± standard deviation, 3.57 ± 1.85 s for the children with ASD and 3.36 ± 1.66 s for those with TD; Japanese class: 3.14 ± 1.70 s for the children with ASD and 3.82 ± 1.38 s for those with TD; F _(1, 51)_ = .42, *p* = .522, two-way ANOVA). To analyze the duration of attention to the teacher and his instructions in the classroom scenes, we calculated the proportion of total viewing time that the children spent gazing at the defined ROIs (Fig 3). Both children with ASD and those with TD looked at the figures pointed at by the teacher for the longest period and at the indicators (the teacher’s face and finger) for the second longest period.

We further analyzed the mean proportion of total gaze time for all movies in each class subject directed to each ROI by a two-way ANOVA with group of children (ASD and TD) as the between factor and class subject (Japanese and arithmetic) as the within factor.

#### Figures pointed at by the teacher

As shown in Fig 3A, the proportion of gaze time spent on the figures that the teacher pointed at was significantly different between the groups of children (F _(1, 51)_ = 5.78, *p* = .020) and the class subjects (F _(1, 51)_ = 13.82, *p* = .001), with interaction between these factors (F _(1, 51)_ = 5.01, *p* = .030). Children with ASD spent significantly less time looking at the cartoon figures in the Japanese classroom scenes than did those with TD (t _(43)_ = 6.91, *p* = .012), but the proportion of gaze time that they spent on the geometric figures in the arithmetic class was not significantly different from that for children with TD (t _(48)_ = .004, *p* = .952).

#### Face and fingers of the teacher

Next, we compared the proportion of gaze time spent on the teacher’s face and fingers. Both the children with ASD and those with TD spent more time looking at the teacher’s face in the Japanese classroom scenes than in the arithmetic classroom scenes (F _(1, 51)_ = 16.66, *p* < .001), but there was no significant difference between ASD and TD children (F _(1, 51)_ = .24, *p* = .624) (Fig 3B).

The proportion of gaze time spent on the teacher’s fingers significantly differed between ASD and TD children (F _(1, 51)_ = 9.69, *p* = .003) and between class subjects (F _(1, 51)_ = 24.03, *p* < .001), with interaction between these factors (F _(1, 51)_ = 8.44, *p* = .005) (Fig 3C). Children with ASD spent less time looking at the teacher’s fingers than did those with TD in the arithmetic classroom scenes (t _(39)_ = 10.19, *p* = .003), but not in the Japanese classroom scenes (t _(51)_ = .31, *p* = .578).

#### Wall

We also analyzed gaze time spent on the wall where no relevant objects existed (shaded with red in Fig 1C and 1D). A long gaze time on the wall indicates that the children were not following the teacher’s instructions. As shown in Fig 3D, the proportion of gaze time spent on the wall significantly differed between ASD and TD children (F _(1, 51)_ = 8.26, *p* = .006), but not between class subjects (F _(1, 51)_ = .54, *p* = .467), with interaction between these factors (F _(1, 51)_ = .09, *p* = .760). Children with ASD spent significantly more time than children with TD looking at the wall, which was irrelevant to the classroom lesson, in both classroom scenes (Fig 3D).

### Joint attention

**Fig 4:**
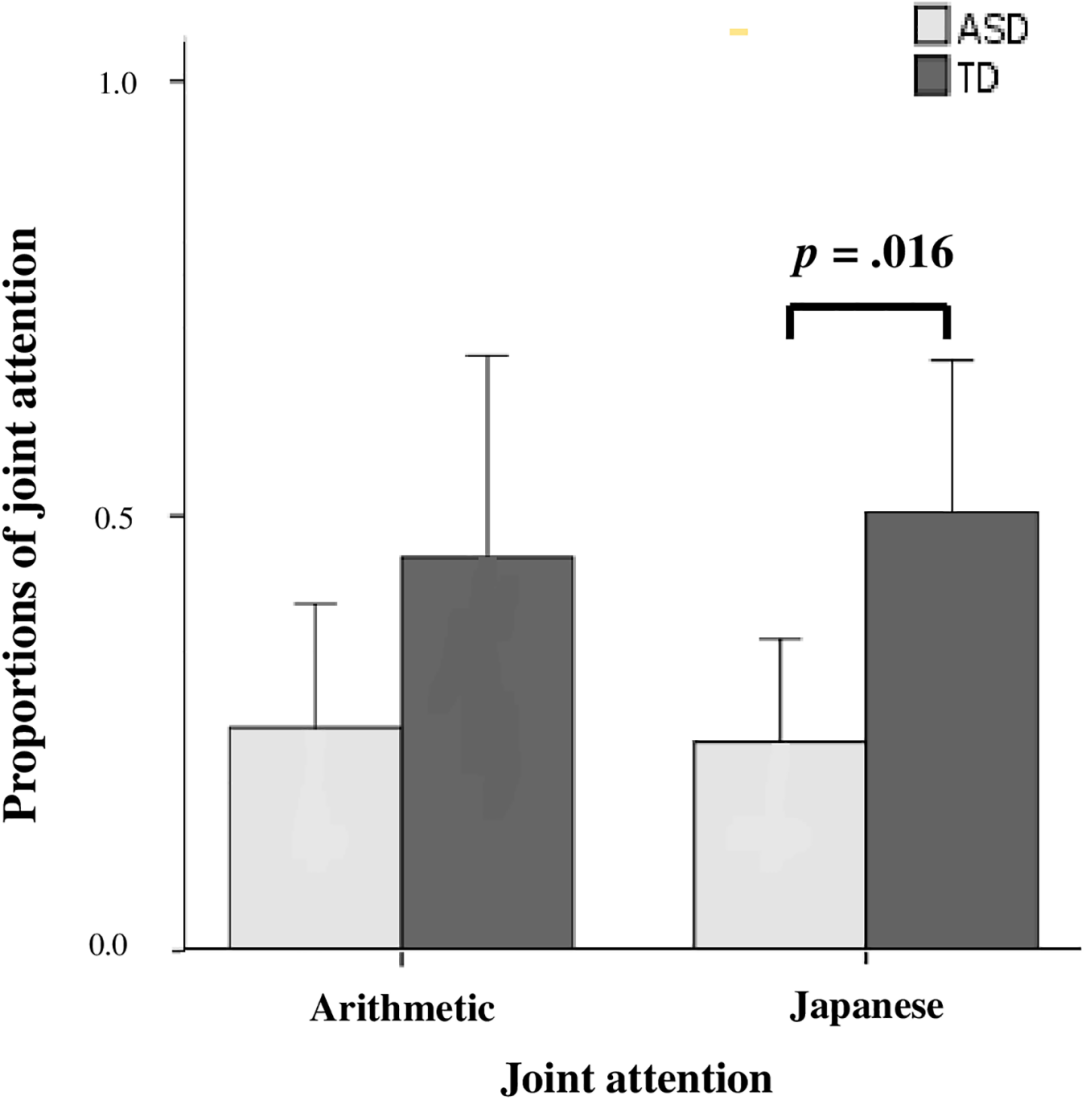
Proportions of frequency of joint attention for the two groups in each classroom scene. The proportions of the frequency of joint attention for the two groups (mean ± standard error of mean) in the arithmetic and Japanese classroom scenes are shown. Gray bars indicate children with ASD, and dark gray bars indicate children with TD. *P* values were determined by Welch’s *t*-test.

The preceding analysis compared the gaze time spent on each ROI by children with ASD and TD. We next analyzed the time course of the gaze behaviors: whether the children looked at the object after looking at the teacher, which is known as “joint attention” [23, 24]. We defined the proportion of joint attention as the number of times a child gazed at a figure that the teacher was pointing to or looking at after gazing at the teacher’s face and/or fingers relative to the total number of gazes at figures. A two-way ANOVA revealed that the proportion of frequency of joint attention was significantly less in children with ASD than in those with TD in both Japanese and arithmetic classes (F _(1, 44)_ = 5.56, *p* = .023) (Fig 4).

### First gaze

**Fig 5:**
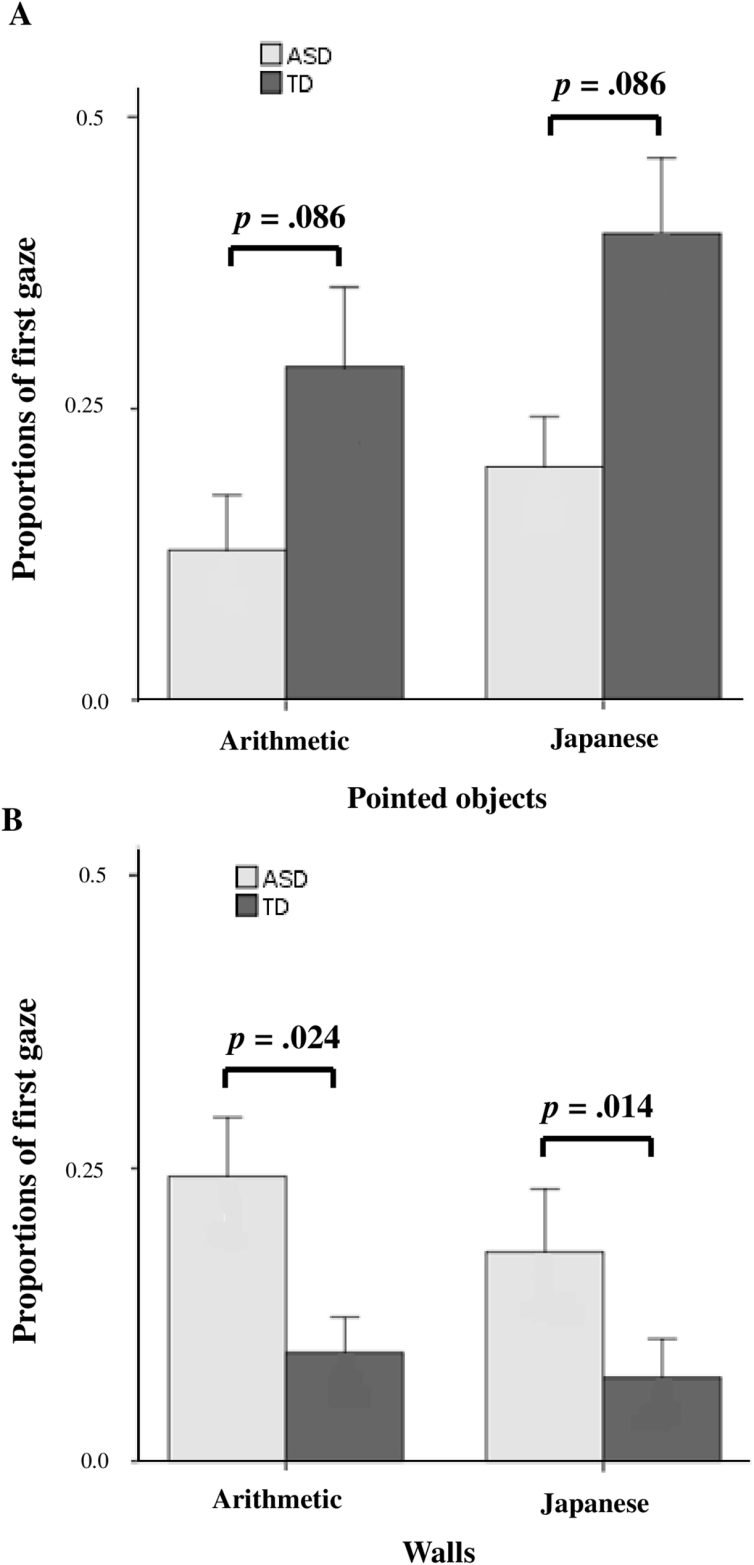
Proportions of the first gaze on the figures pointed at by the teacher and on the wall in each classroom scene. (A) Proportions of the first gaze on the geometric figures pointed at by the teacher in the arithmetic classroom scenes and on the cartoon figures in the Japanese classroom scenes. (B) Proportions of the first gaze spent on the wall in both classroom scenes. Gray bars indicate children with ASD, and dark gray bars indicate children with TD. *P* values were determined by Welch’s *t*-test.

The analysis so far has focused on how long the children looked at the relevant parts of the scene during the whole period when they were watching the movies. We next asked whether there was a significant difference between the ASD and TD groups in the direction of the gaze at the beginning of each video scene (“first gaze”) (Fig 5). The two-way ANOVA revealed that there were significant differences between the groups in the proportion of the first gaze on the objects at which the teacher was pointing (F _(1, 44)_ = 4.56, *p* = .038) (Fig 5A) and in the proportion of the first gaze on the wall (F _(1, 44)_ = 15.52, *p* < .001) (Fig 5B). More children with ASD than those with TD directed their first gaze to the wall. These results indicate that children with ASD directed their first gaze to the inappropriate space of the video, i.e., the wall, rather than to the teacher, and spent more time looking at the inappropriate space. The children with TD, on the other hand, started by looking at the teacher, and then, looked at the objects at which he was pointing.

### Developmental effect

**Fig 6:**
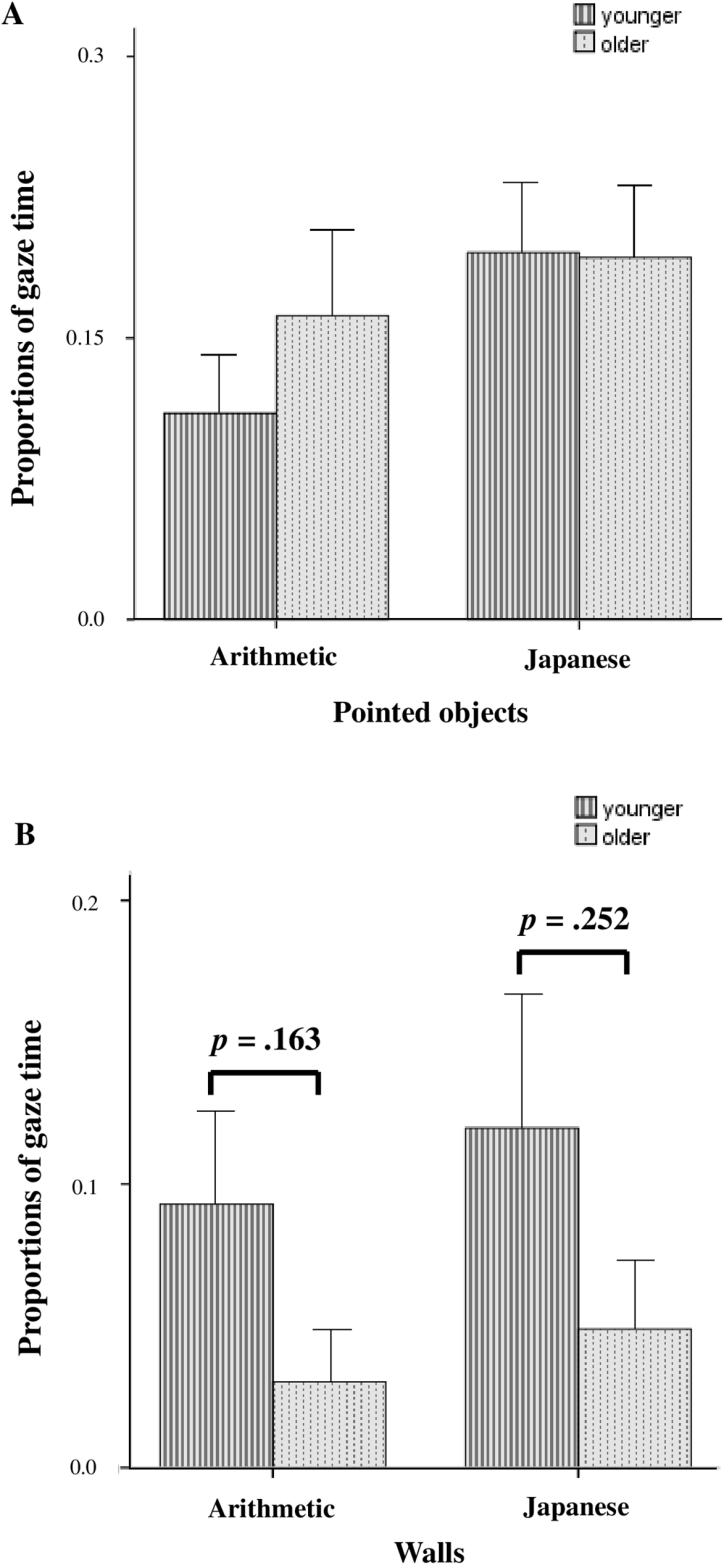
Developmental effect on proportions of gaze time in children with ASD. (A) Proportions of gaze time spent on the geometric figures directed by the teacher in the arithmetic class and the cartoon figures in the Japanese classroom scenes. (B) Proportions of gaze time spent on inappropriate visual areas (walls) in both classroom scenes. Stripped gray bars indicate children with ASD (2 to 9 years old), and dotted gray bars indicate adolescents with ASD (10 to 15 years old). *P* values were determined by Welch’s *t*-test.

We also analyzed the effect of development on gaze characteristics of the children with ASD and TD. We divided the participants into two groups consisting of children under 10 years of age and those 10 years of age or older. The age of 10 is thought to be a threshold of development, according at which children 10 to 15 years of age are capable of formal reasoning, whereas those under 10 are still at the concrete level [25]. We directly compared younger (2 to 9 years) and older (10 to 15 years) children in the proportion of gaze time they spent on the figures pointed at by the teacher and the wall (Fig 6). As shown in Fig 6A, there was no significant difference between the younger and older children with ASD in the amount of gaze time spent on the figures pointed at by the teacher (F _(1, 24)_ = 2.10, *p* = .161 for the age groups). In contrast, there was a marginal difference between the younger and older children with ASD in the amount of gaze time spent on the wall (F _(1, 24)_ = 3.28, *p* = .083 for the age groups). The younger children with ASD spent more time looking at the inappropriate area (the wall) than the older children with ASD (Fig 6B).

Likewise, there was no significant difference between the younger and older children with TD in the amount of gaze time spent on the figures pointed at by the teacher (F _(1, 25)_ = .135, *p* = .717 for the age groups). In contrast, the younger children with TD spent more time looking at the inappropriate area (the wall) than the older children with TD (F _(1, 25)_ = 4.64, *p* = .041 for the age groups).

Thus, the children with ASD developed more slowly to be able to locate appropriate areas to understand the other people’s thoughts than those with TD.

### Application as a screening and support tool for children with ASD

**Fig 7:**
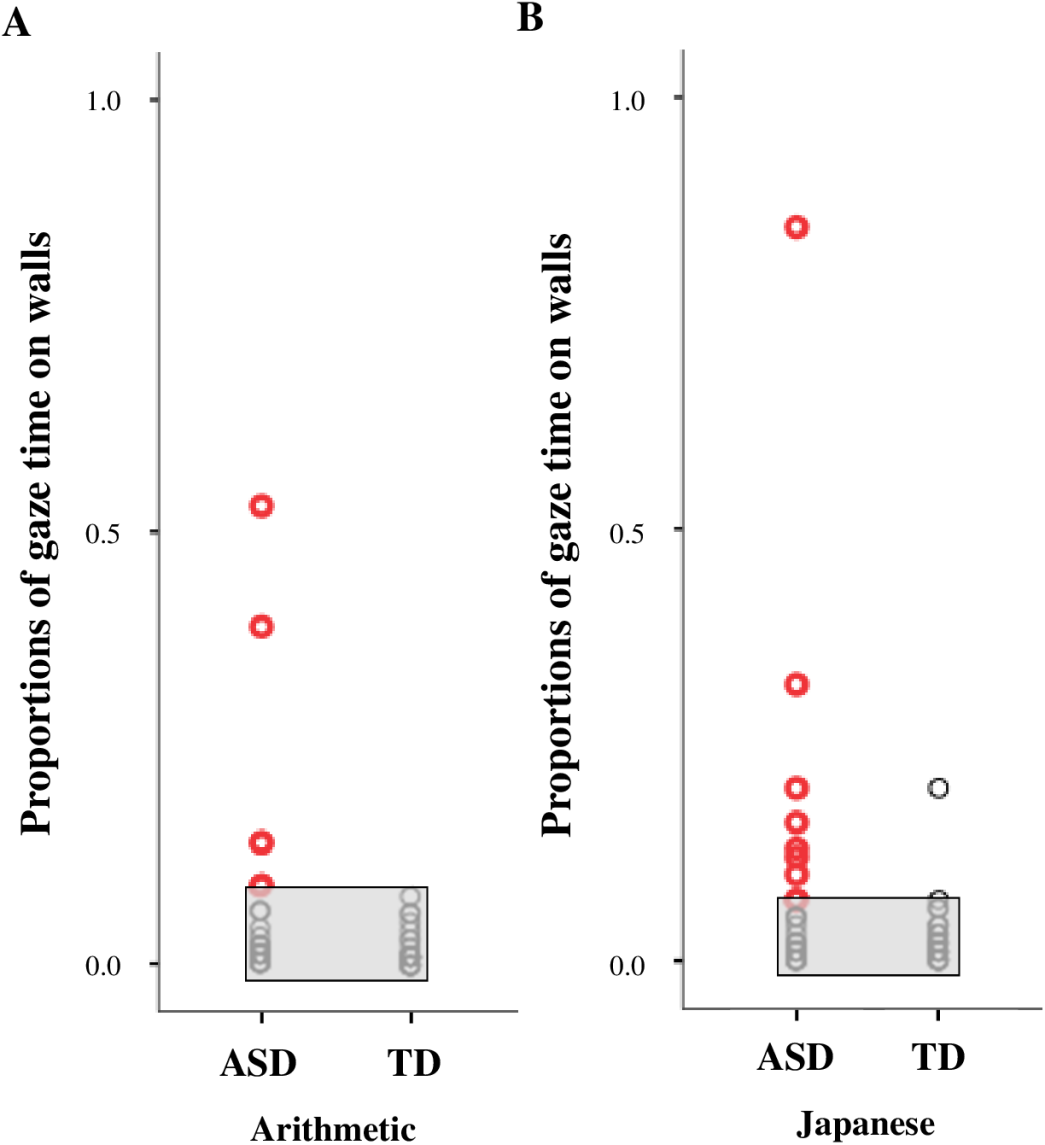
Scatterplot illustrating the proportions of gaze time spent on the wall in each classroom scene. (A, B) Proportions of gaze time spent on the wall in the arithmetic and Japanese classroom scenes for the two groups. When the proportion of gaze time that a child spent on the wall was more than 0.09 in the arithmetic or the Japanese classroom scene, we could classify the child as having ASD with an accuracy of almost 100%.

Finally, we evaluated the applicability of the proportion of gaze time spent on the wall as a screening tool for children with ASD. As shown in Fig 7, we found that children who spent more than 0.09 of their proportion of gaze time looking at the wall in either the Japanese or the arithmetic classroom scene could be accurately diagnosed with ASD (*p* = .022 for the Japanese class, *p* = .010 for the arithmetic class; Fisher’s exact test).

In some situations in both the Japanese and arithmetic classroom scenes, the children with ASD spent more time looking at objects at which the teacher pointed by a “congruent” gaze than objects indicated by either a “direct” gaze or an “incongruent” gaze. Moreover, in the Japanese classroom scenes, children with ASD spent more time looking at objects at which the teacher pointed when the teacher was far from the objects at which he was pointing than when he was near the objects.

### Test–retest reliability

To ascertain the consistency of the gaze characteristics across different experimental sessions, five children (one with ASD and four with TD) returned for a second eye-tracking session 1 to 2 years following the first session. Test–retest reliability was analyzed by the proportion of gaze time spent on each ROI (the objects at which the teacher was pointing, the wall, the teacher’s face, and the teacher’s fingers) in the arithmetic and Japanese classroom scenes between the first and second sessions. A paired-sample *t-*test revealed no significant differences between the first and second sessions for any ROI (t _(4)_ = −1.22, *p* = .291, t _(4)_ = 1.62, *p* = .180 for the objects at which the teacher was pointing in the arithmetic and Japanese classes; t _(4)_ = −1.20, *p* = .296, t _(4)_ = −.54, *p* = .621 for the wall in the arithmetic and Japanese classes; t _(4)_ = −2.24, *p* = .089, for the teacher’s face in the arithmetic class; t _(4)_ = .00, *p* = 1.00, t _(4)_ = −1.50, *p* = .208 for the teacher’s fingers in the arithmetic and Japanese classes) except for the teacher’s face in the Japanese class (t _(4)_ = −3.00, *p* = .040).

## Discussion

In the present study, we investigated the characteristics of the gaze behavior of children with ASD and TD when they viewed movies of actual school classroom scenes. The children with ASD spent less time looking at the cartoon figures pointed at by the teacher and more time looking at the wall than did children with TD. They also showed less joint attention than children with TD. These characteristics of the gaze of children with ASD can be applied for a screening or diagnostic tool for children with ASD. This study has demonstrated the validity of actual classroom scenes as visual stimuli in studies of the gaze behavior of children with ASD.

### Where and how did the children with ASD gaze?

#### The teacher’s face

The children with ASD spent as much time looking at the teacher’s face as did the children with TD. This is inconsistent with previous reports that children with ASD spent less gaze time on the human face than did those with TD [26, 27]. Irwin and Brancazio [28] reported that children with ASD looked at the speaker’s face less and fixated on the speaker’s mouth less than did children with TD. However, when the speaker was pointing to something, school-aged children with ASD spent as much time gazing at the speaker’s face as did those with TD [29]. In addition, children with ASD could spend more time looking at the human face and eyes when they were instructed to detect and name what others were looking at [12]. In the current study, since the teacher pointed at and/or looked at the figures in each situation, the children with ASD gazed at the teacher’s face for as long as those with TD. Riby and Hancock [30] reported that participants with autism showed a significant negative correlation between level of functioning according to CARS and the amount of time spent looking at faces; thereby, longer face gaze was associated with higher functioning on the autism spectrum. The children with ASD in our study had low CARS scores and looked at the teacher’s face as long as did children with TD, a result similar to that of Riby and Hancock. Thus, these results revealed that high-functioning children with ASD looked at the human face as long as did children with TD.

However, further analyses in our study revealed that children with ASD had less joint attention and took more time to look at the objects at which the teacher was pointing than did those with TD. They also spent more time looking at the wall than did children with TD. Taking together, the results suggest that children with ASD have difficulty in recognizing the objects at which the teacher is pointing, even if they spend as much time gazing at the teacher’s face as do those with TD.

#### Objects at which the teacher was pointing and joint attention

In our study, the duration of gaze on the geometric figures pointed at by the teacher was not significantly different between the ASD and TD groups, a result that is inconsistent with those of Pierce and colleagues [9, 10] that children with ASD spend significantly more gaze time on dynamic geometric images than do those with TD. In addition, the children with ASD spent more time than did those with TD looking at the wall, which was not pointed at by the teacher, even in the arithmetic classroom scenes with geometric figures. These results suggest that children with ASD do not necessarily prefer looking at geometric figures in actual classroom scenes. They may direct their attention to irrelevant areas because of failure of joint attention [31, 32], since their joint attention was less than that of children with TD (Fig 4).

Another possibility is that they prefer simpler visual areas such as walls, with lower contrast in color, luminance, and texture [33, 34]. They may try to avoid looking at the teacher and the other students, and consequently, their eyes may often land on the irrelevant visual areas where people and figures are not included. This is in agreement with the reports of Shic et al. [35] and Riby and Hancock [36] that children with ASD tend to look at the background in communication scenes and animations without geometric images.

Although we need further studies to understand what determines gaze behavior in children with ASD, the actual classroom stimuli that we used were efficacious in differentiating children with ASD from those with TD and can be applied to a new screening or diagnostic tool. When testing children’s responses to visual stimuli, inclusion of an area such as a wall with few distractors and low contrast in color, luminance, and texture may play an important role in diagnosing ASD.

### Future application as a screening and support tool for children with ASD

On the basis of these gaze characteristics, we considered how we could detect and support children with ASD. First, the differences in gaze characteristics between children with ASD and those with TD can be applied to a tool for screening children for ASD. By determining the proportion of gaze time spent looking at the wall, we can accurately differentiate between children with ASD and those with TD. In both the Japanese and arithmetic classroom scenes, the proportion of gaze time spent on looking at the wall by children with ASD was more than 0.09. Similarly, although we need further analyses, the differences between children with ASD and those with TD in the amount of joint attention and the direction of the first gaze can be applied to a tool for screening children with ASD.

Second, the children with ASD spent more time looking at objects at which the teacher pointed by a “congruent” gaze than objects indicated by either a “direct” gaze or an “incongruent” gaze. This suggests that the children with ASD can gaze at the appropriate visual areas pointed at by the teacher, even if they have weak joint attention ability. Moreover, in the Japanese classroom scenes, children with ASD spent more time looking at objects at which the teacher pointed when the teacher was far from the objects at which he was pointing than when he was near the objects. Although this result is counterintuitive, it can be explained by the reasons described above: when the teacher was far, the visual areas were simpler and had lower contrast and it was easier for the children to avoid the teacher. Around the figures pointed at by the teacher, there were fewer distractors with longer distances among figures, indicating simpler and lower contrast in color, luminance, and texture, so that their eyes might more frequently land on the appropriate visual areas, including the figures pointed at by the teacher. Such visual areas were nearer than the other visual areas of the inappropriate areas—lower cost in action.

These findings from children with ASD may help to improve their understanding of content in class. For instance, when teachers want students with ASD to gaze at something drawn or written on the blackboard, they can direct their attention by both looking at it and pointing to it, without touching it.

### Limitations

There are several limitations of this study. First, we used the actual classroom scenes as visual stimuli in which the teacher pointed at the geometric figures in the arithmetic and the cartoon figures in the Japanese classroom scenes. However, we should further investigate other situations in which the teacher points not to the figures but to the Japanese characters or numbers. Second, we did not investigate the children’s understanding of the content of the objects at which the teacher was pointing. Although we found that children with ASD spent less time looking at the objects at which the teacher was pointing and exhibited less joint attention than did those with TD, we should investigate the degree of understanding of the objects at which the teacher was pointing to support our findings. Third, we did not investigate the proportion of gaze time spent on the teacher’s eyes and mouth because of the small size of these stimuli. Last, it is essential to further investigate how to determine an early diagnosis using a larger sample of toddlers as well as to ascertain the relations between gaze characteristics and understanding school classes using a larger sample of adolescents.

In summary, we found that analysis of the gaze behavior of children with ASD in actual classroom scenes can be applied not only as a screening tool for ASD but also as a support tool for the children to improve their understanding of content in class.

## Supporting information

S1 Fig Proportions of gaze time on the geometric figures pointed at by the teacher in the arithmetic class. Scatterplots of all participants illustrating the proportion of total viewing time for all movies in the arithmetic class on the geometric figures pointed at by the teacher.
(EPS)

S2 Fig Proportions of gaze time on the cartoon figures pointed at by the teacher in the Japanese class. Scatterplots of all participants illustrating the proportion of total viewing time for all movies in the Japanese class on the cartoon figures pointed at by the teacher.
(EPS)

S3 Fig Proportions of gaze time on the teacher’s face in the arithmetic class. Scatterplots of all participants illustrating the proportion of total viewing time for all movies in the arithmetic class on the teacher’s face.
(EPS)

S4 Fig Proportions of gaze time on the teacher’s face in the Japanese class. Scatterplots of all participants illustrating the proportion of total viewing time for all movies in the Japanese class on the teacher’s face.
(EPS)

S5 Fig Proportions of gaze time on the teacher’s fingers in the arithmetic class. Scatterplots of all participants illustrating the proportion of total viewing time for all movies in the arithmetic class on the teacher’s fingers.
(EPS)

S6 Fig Proportions of gaze time on the teacher’s fingers in the Japanese class. Scatterplots of all participants illustrating the proportion of total viewing time for all movies in the Japanese class on the teacher’s fingers.
(EPS)

S7 Fig Proportions of gaze time on the walls in the arithmetic class. Scatterplots of all participants illustrating the proportion of total viewing time for all movies in the arithmetic class on the walls.
(EPS)

S8 Fig Proportions of gaze time on the walls in the Japanese class. Scatterplots of all participants illustrating the proportion of total viewing time for all movies in the Japanese class on the walls.
(EPS)

S9 Fig The joint attention in the arithmetic class. Scatterplots of all participants illustrating the proportion of total joint attention for all movies in the arithmetic class.
(EPS)

S10 Fig The joint attention in the Japanese class. Scatterplots of all participants illustrating the proportion of total joint attention for all movies in the Japanese class.
(EPS)

S11 Fig The first gaze on the geometric figures pointed at by the teacher in the arithmetic class. Scatterplots of all participants illustrating the proportion of total first gaze for all movies in the arithmetic class on the geometric figures pointed at by the teacher.
(EPS)

S12 Fig The first gaze on the cartoon figures pointed at by the teacher in the Japanese class. Scatterplots of all participants illustrating the proportion of total first gaze for all movies in the Japanese class on the cartoon figures pointed at by the teacher.
(EPS)

S13 Fig The first gaze on the walls in the arithmetic class. Scatterplots of all participants illustrating the proportion of total first gaze for all movies in the arithmetic class on the walls.
(EPS)

S14 Fig The first gaze on the walls in the Japanese class. Scatterplots of all participants illustrating the proportion of total first gaze for all movies in the Japanese class on the walls.
(EPS)

S15 Fig The developmental effect in the participants with ASD on the geometric figures pointed at by the teacher in the arithmetic class. Scatterplots of all participants with ASD illustrating the proportion of total viewing time for all movies in the arithmetic class on the geometric figures pointed at by the teacher.
(EPS)

S16 Fig The developmental effect in the participants with ASD on the cartoon figures pointed at by the teacher in the Japanese class. Scatterplots of all participants with ASD illustrating the proportion of total viewing time for all movies in the Japanese class on the cartoon figures pointed at by the teacher.
(EPS)

S17 Fig The developmental effect in the participants with ASD on the walls in the arithmetic class. Scatterplots of all participants with ASD illustrating the proportion of total viewing time for all movies in the arithmetic class on the walls.
(EPS)

S18 Fig The developmental effect in the participants with ASD on the walls in the Japanese class. Scatterplots of all participants with ASD illustrating the proportion of total viewing time for all movies in the Japanese class on the walls.
(EPS)

S19 Fig The developmental effect in the participants with TD on the geometric figures pointed at by the teacher in the arithmetic class. Scatterplots of all participants with TD illustrating the proportion of total viewing time for all movies in the arithmetic class on the geometric figures pointed at by the teacher.
(EPS)

S20 Fig The developmental effect in the participants with TD on the cartoon figures pointed at by the teacher in the Japanese class. Scatterplots of all participants with TD illustrating the proportion of total viewing time for all movies in the Japanese class on the cartoon figures pointed at by the teacher.
(EPS)

S21 Fig The developmental effect in the participants with TD on the walls in the arithmetic class. Scatterplots of all participants with TD illustrating the proportion of total viewing time for all movies in the arithmetic class on the walls.
(EPS)

S22 Fig The developmental effect in the participants with TD on the walls in the Japanese class. Scatterplots of all participants with TD illustrating the proportion of total viewing time for all movies in the Japanese class on the walls.
(EPS)
